# Supplementary material for: Adaptation of the Endolithic Biome in Antarctic Volcanic Rocks
Source: Int J Mol Sci. 2023 Sep 7;24(18):13824. doi: 10.3390/ijms241813824 (PMC10530270; doi:10.3390/ijms241813824)
Supplement: Supplementary file 1 [file ijms-24-13824-s001.zip › ijms-2559347-supplementary.pdf]

International Journal of
Molecular Sciences

SUPPLEMENTARY INFORMATION FOR:

Adaptation of the endolithic biome in Antarctic volcanic rocks

Andrea Hidalgo-Arias [1,†], Víctor Muñoz-Hisado [1,†], Pilar Valles [2], Adelina Geyer [3], Eva Garcia-Lopez [1] and Cristina Cid [1,*]

[1] Center for Astrobiology (CAB), CSIC-INTA, 28850 Torrejón de Ardoz, Madrid, Spain; andhid01@ucm.es (A.H.-A.); vmhisado@cab.inta-csic.es (V.M.-H.); evanit3@hotmail.com (E.G.-L.)
[2] Materials and Structures Department, National Institute of Aerospace Technology (INTA), 28850 Torrejón de Ardoz, Madrid, Spain; vallesgp@inta.es
[3] Geosciences Barcelona (GEO3BCN), CSIC, Lluís Solé Sabarís s/n, 08028 Barcelona, Spain; ageyer@geo3bcn.csic.es
* Correspondence: cidsc@inta.es or cidsc@cab.inta-csic.es; Tel.: +34-91-520-5455
† These authors contributed equally to this work.

This file includes:
I. Supplementary Tables. S1–S7.
II. Supplementary Figures. S1–S10.

I. Supplementary Tables

Table S1. Elemental concentrations in volcanic rocks.

Sample	C	Na	Mg	Al	Si	P	S	Cl	K	Ca	Cr	Mn	Fe	Zn
NB1_S2a	452	27940	5575	27281	96013	269	1218	18373	4364	36293	52	1342	70839	87
NB1_S3b	444	28456	5423	25698	90236	201	1587	16320	4012	33654	50	1265	87569	80
NB1_S4c	463	26321	5699	24187	88654	200	1698	15478	3987	32014	51	1125	88965	77
DIPV36_S3a	368	30821	7223	33588	100470	304	1014	16343	4402	44615	46	1583	84237	100
DIPV36_S4b	401	31297	7165	30214	92541	299	998	14569	4412	45236	39	1547	99875	100
DIPV36_S5c	299	29875	7366	30225	90214	298	965	12365	4563	49875	36	1658	99965	120
DIVOL_12Aa	568	35750	7396	41798	112179	339	513	18030	6504	43222	65	1580	75923	100
DIVOL_12Ab	574	36332	7456	44129	123654	356	321	20156	6654	45236	70	1581	60214	150
DIVOL_12Ac	554	34587	7211	43012	125874	362	365	19874	6785	44216	71	1588	59654	110
DIVOL_4Aa	40	2269	3955	11547	30654	85	141	20143	7021	51021	10	125	20325	54
DIVOL_4Ab	38	2134	4021	10236	31254	80	99	21023	6987	50214	11	123	18654	55
DIVOL_4Ac	41	2265	3865	10254	31265	77	182	22365	6847	51236	12	122	17458	49
DIVOL_23a	45	44593	5400	10325	39693	87	12	40215	10547	55036	15	236	10254	39
DIVOL_23b	44	51478	5142	10254	40215	79	15	45879	11478	54123	12	251	9874	38
DIVOL_23c	63	49652	5231	10365	41239	81	19	19654	12365	53240	11	220	9965	36

*BD: below detection; the highest nutrient values are marked in bold. Concentrations are expressed in ppm (a, b and c represent 3 replicates).

Table S2. Correspondence analyses and correlations (λ).

No. of analysis	Type of microorganism	Level	Type of analysis	Variables	λ_1	λ_2	λ_3	λ_4	Figure
1	Bacteria	Phylum	PCA	-	0.789	0.176	0.022	0.012	5A
2		Genus	PCA	-	0.957	0.028	0.011	0.003	5B
3		Phylum	CCA	C, Na, Mg, Al, Si, P, S, Cl, K, Ca, Cr, Mn, Fe, Zn	0.186	0.158	0.055	0.021	6A
4		Genus	CCA	C, Na, Mg, Al, Si, P, S, Cl, K, Ca, Cr, Mn, Fe, Zn	0.784	0.248	0.144	0.111	6B
5	Eukarya	Phylum	PCA	-	0.633	0.341	0.016	0.009	5C
6		Genus	PCA	-	0.723	0.247	0.020	0.010	5D
7		Phylum	CCA	C, Na, Mg, Al, Si, P, S, Cl, K, Ca, Cr, Mn, Fe, Zn	0.297	0.086	0.029	0.004	6C
8		Genus	CCA	C, Na, Mg, Al, Si, P, S, Cl, K, Ca, Cr, Mn, Fe, Zn	0.617	0.181	0.061	0.000	6D

Table S3. Adaptive strategies. Common (+) and non-common (-) adaptative strategies between other endolithic biomes and the Antarctic volcanic rock biome identified in this work.

Endolithic biomes	Adaptation	Common adaptation strategies with the Antarctic volcanic rock biome	References
Shallow sedimentary bedrock	Oligotrophic heterotrophs	+	[13]
	Autotrophs using sulfur-based compounds as energy sources	+	[13]
Cave ferromanganese deposits	Fe- and Mn-oxidizing bacteria	+	[64]
Cryptoendolithic habitats	Synthesis of photoprotective-screening and pigments	+	[65-67]
	Production of extracellular polysaccharides (EPS)	+	[66,68]
Deep marine environments	Adaptation to high pressure	-	[69,70]
Cyanobacteria exposed to matric water stress	Resistance to desiccation by accumulating water-stress proteins or producing osmoprotectants such as trehalose and sucrose	?	[71]
Algae and cyanobacteria in cold environments	Control of intracellular ice formation	?	[72]
Cryptoendolithic habitats	Biofilm formation	+	[68]
Volcanic rocks	Adaptation to high temperature	-	[63]

Table S4. Analysis of bacterial 16S rRNA: Genus level.

Table S4.xlsx

Table S5. Analysis of bacterial 16S rRNA: Phylum level.

Table S5.xlsx

Table S6. Analysis of eukaryotic 18S rRNA: Genus level.

Table S6.xlsx

Table S7. Analysis of eukaryotic 18S rRNA: Phylum level.

Table S7.xlsx

II. Supplementary Figures

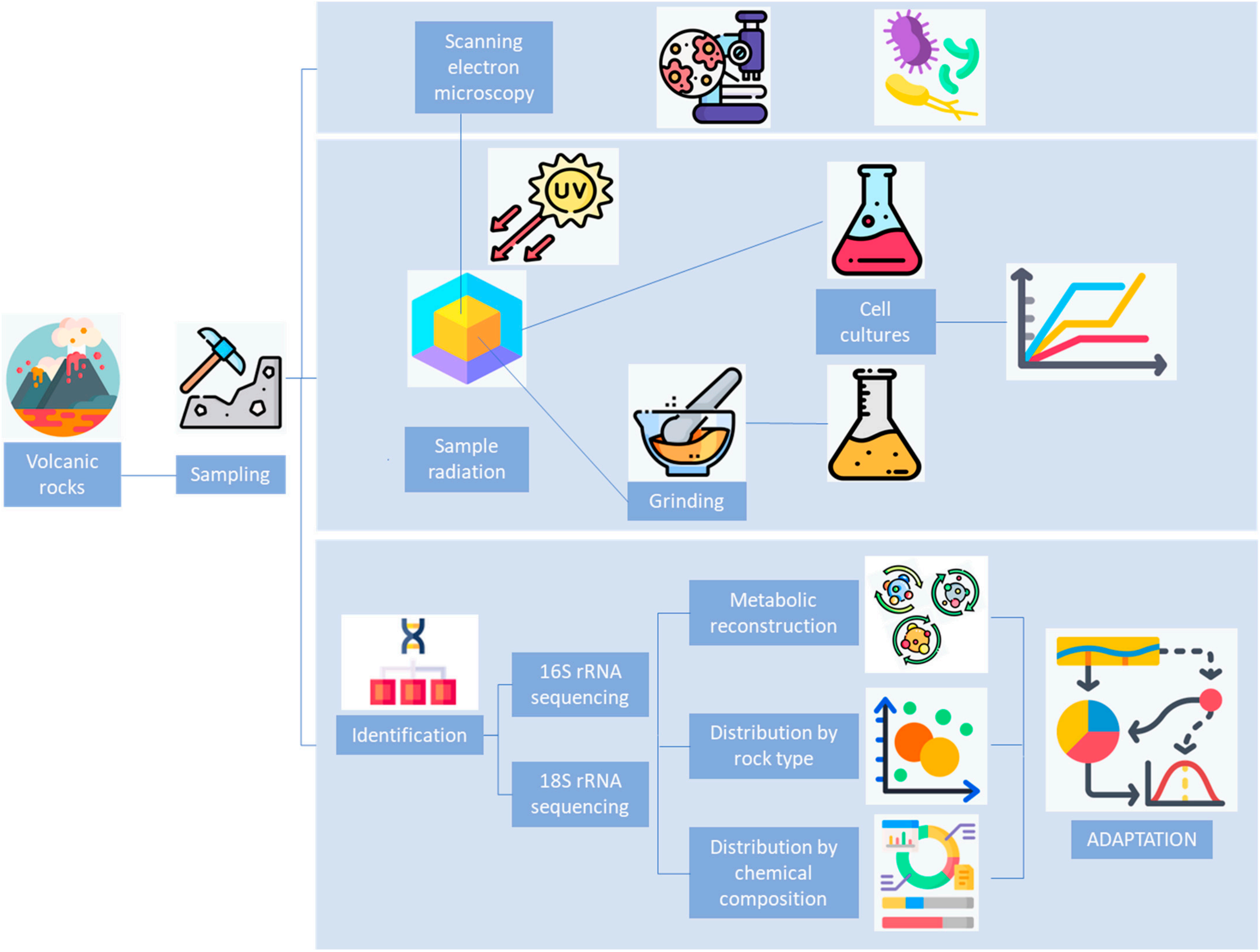

Figure S1. Summary of the overall experimental strategy. Schematic representation of the experimental design followed to identify the endolithic biome in Antarctic volcanic rocks. (Icons are from: https://www.flaticon.es/).

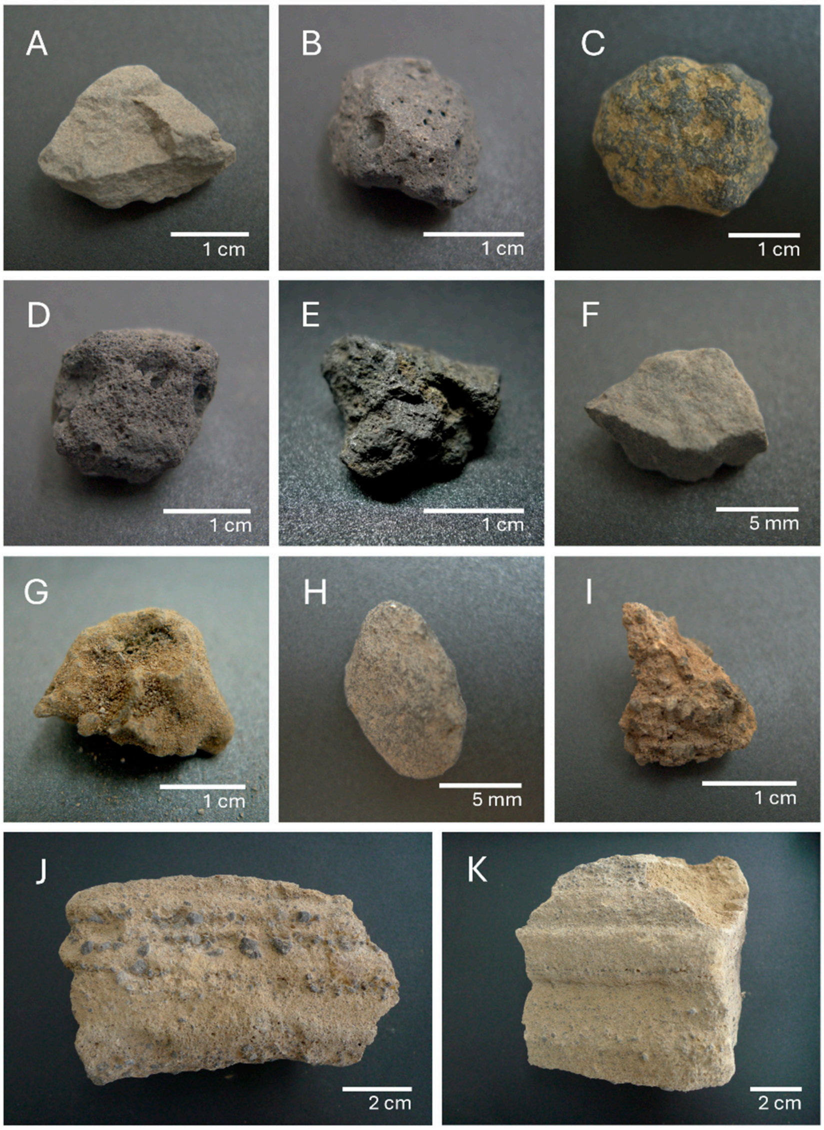

Figure S2. Antarctic volcanic rocks. DIPV36_S1 (A), DIPV36_S2 (B), DIPV36_S3 (C), NB1_S1 (D), NB1_S2 (E), NB1_S3 (F), DIVOL_9 (G), DIVOL_12A (H), DIVOL_23 (I), DIVOL_4A (J) y DIVOL_4D (K). .

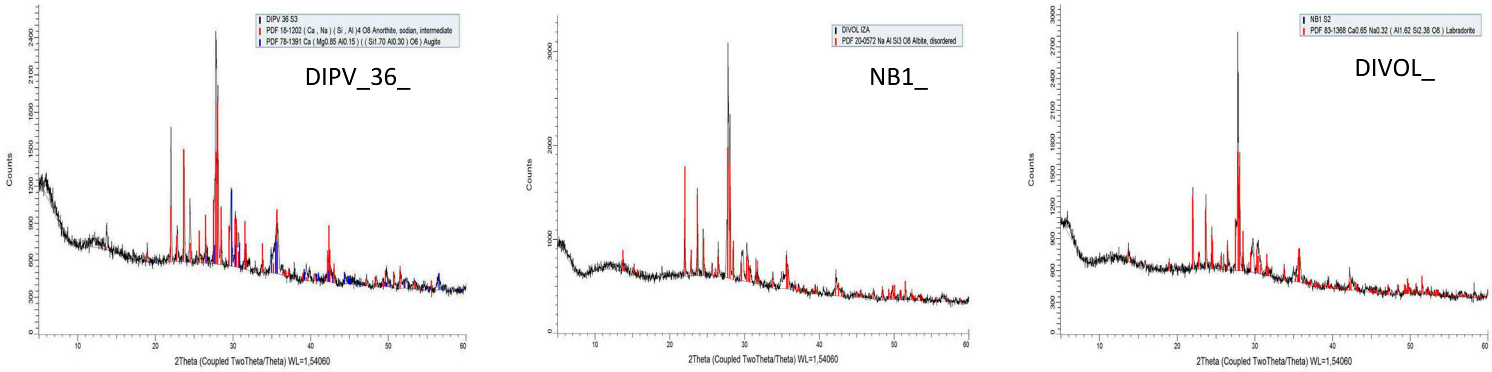

Figure S3. XRD spectra. Examples of spectra from several samples of volcanic rocks analyzed by XRD.

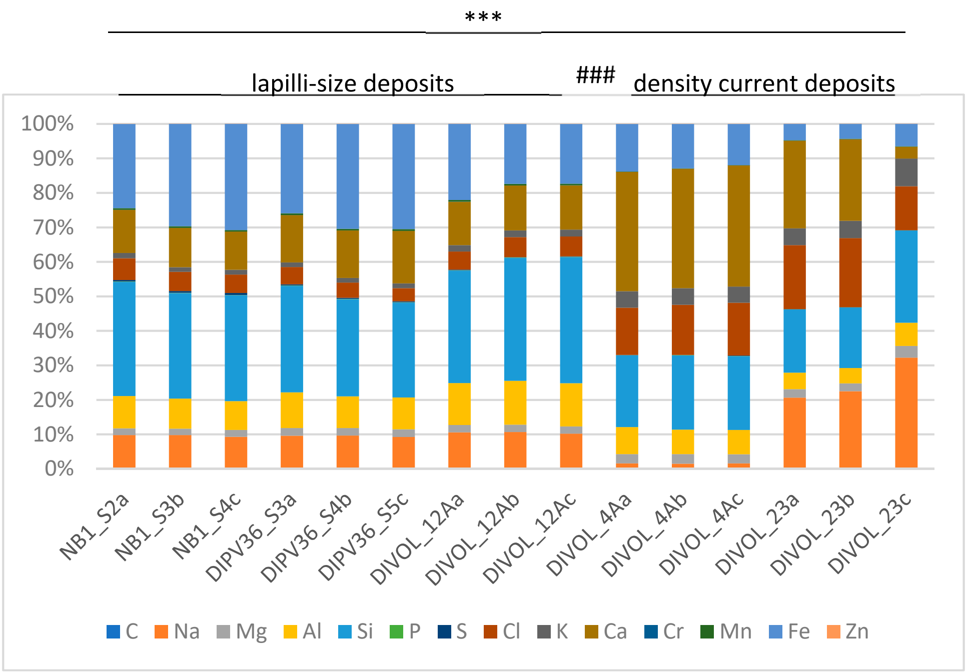

Figure S4. Chemical composition of volcanic rocks. Chemical composition of the two groups of samples: pyroclastic density current deposits versus loose pyroclastic lapilli-size deposits. Significant differences among samples were determined by ANOVA (***$p < 0.0001$). Significant differences between the two groups of samples (pyroclastic density current deposits versus loose pyroclastic lapilli-size deposits) were determined by Student's t-test and Wilcoxon post-test (###$p < 0.0001$).

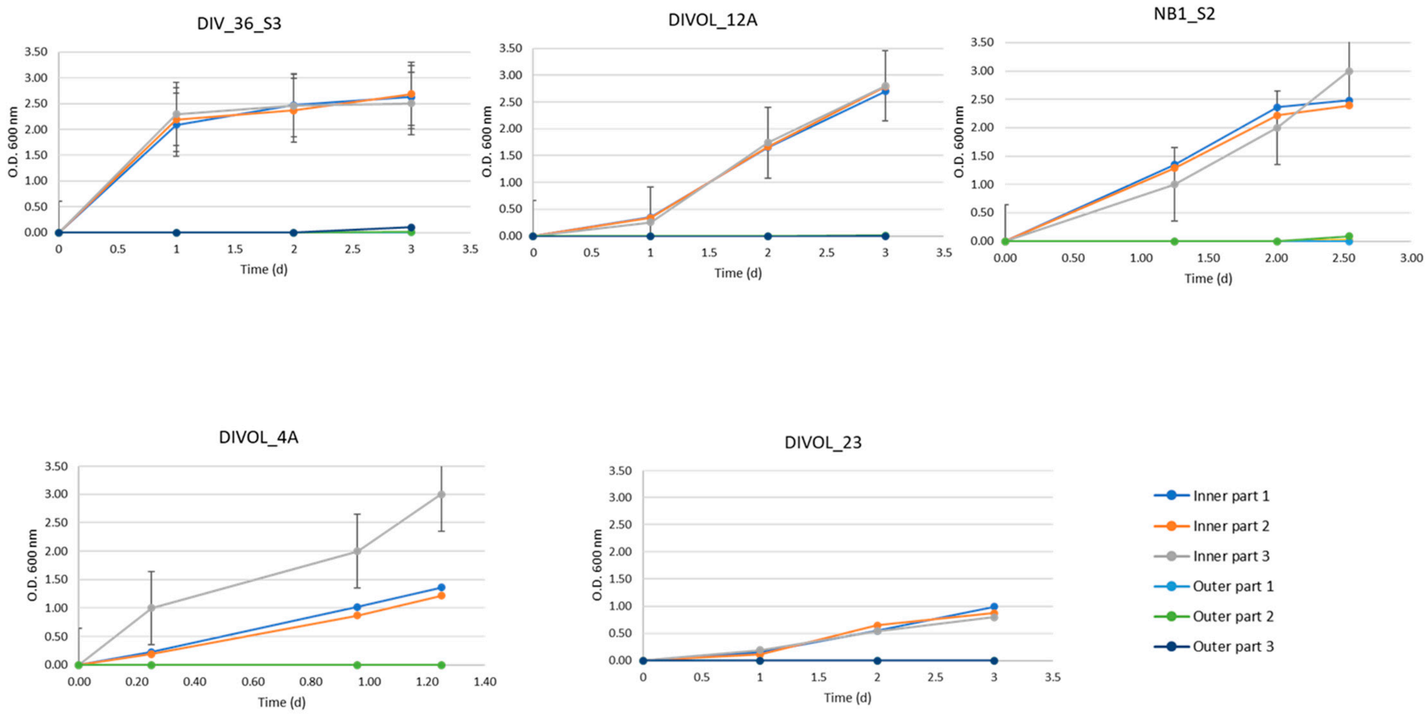

Figure S5. Cell cultures to check endolith viability. Each culture was incubated in R2A medium in triplicate (Inner part 1, 2, 3 and outer part 1, 2, 3).

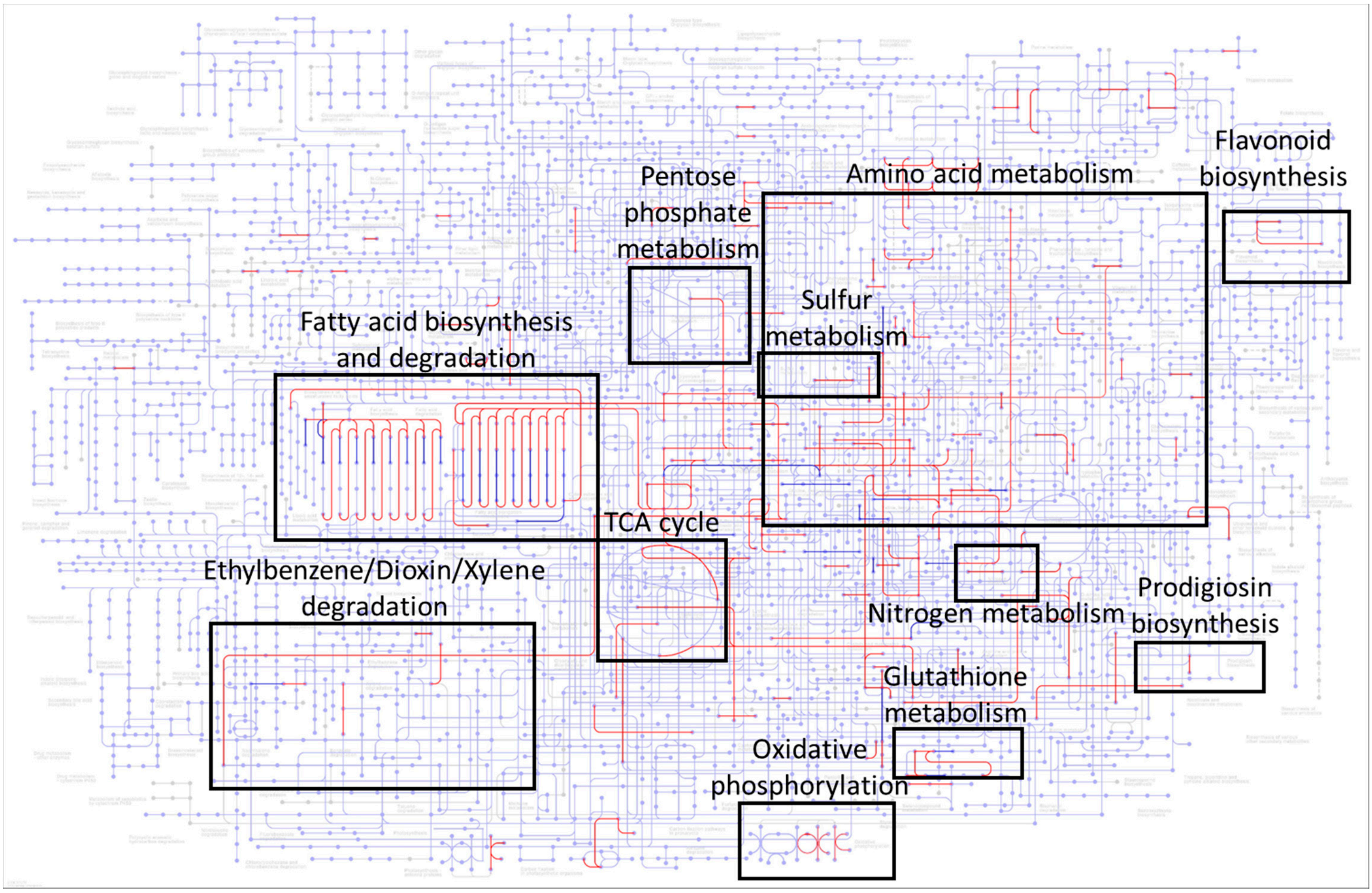

Figure S6. Prediction of metabolic pathways for bacteria in sample DIPV_36_S3.

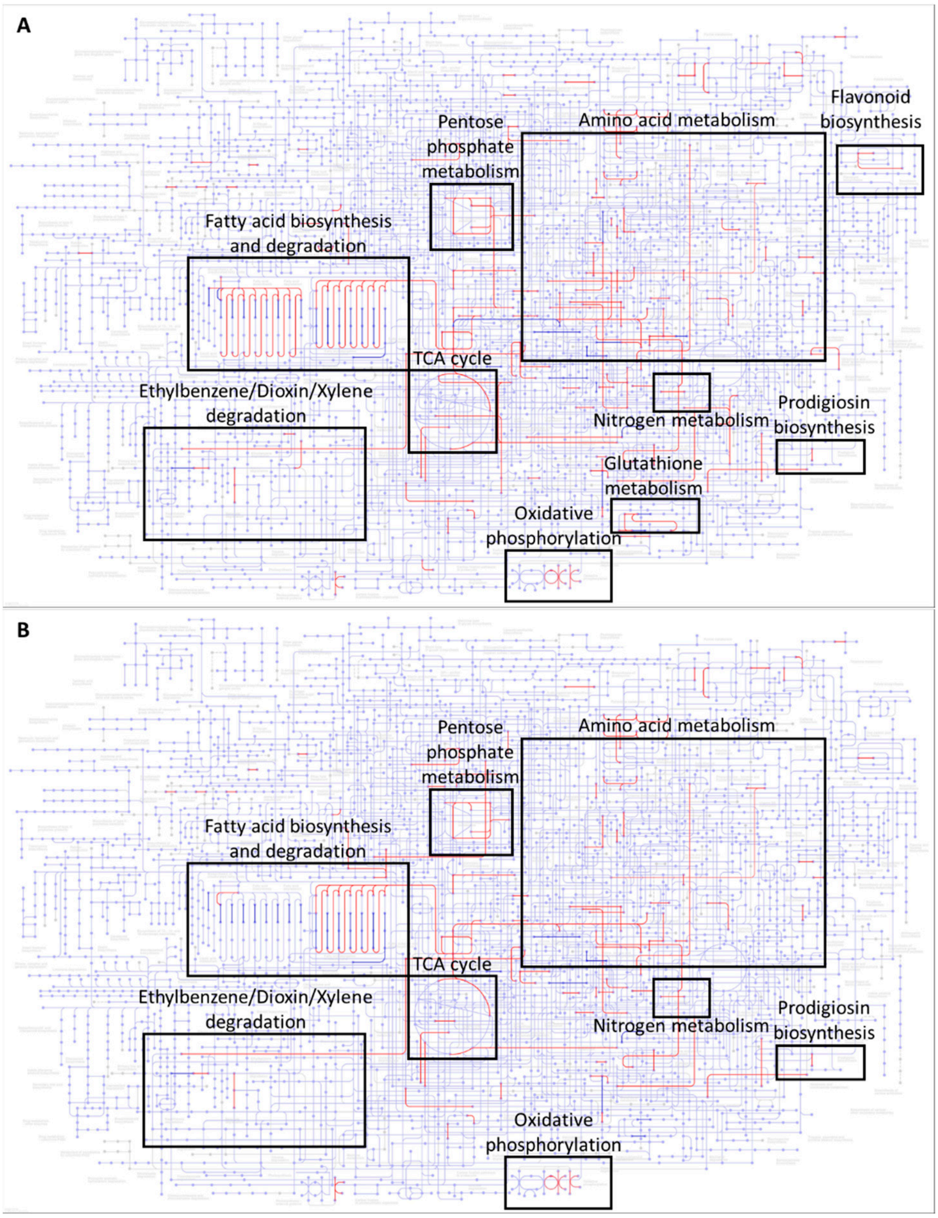

Figure S7. Prediction of metabolic pathways for (A) bacteria and (B) eukaryotes in sample DIVOL_12A.

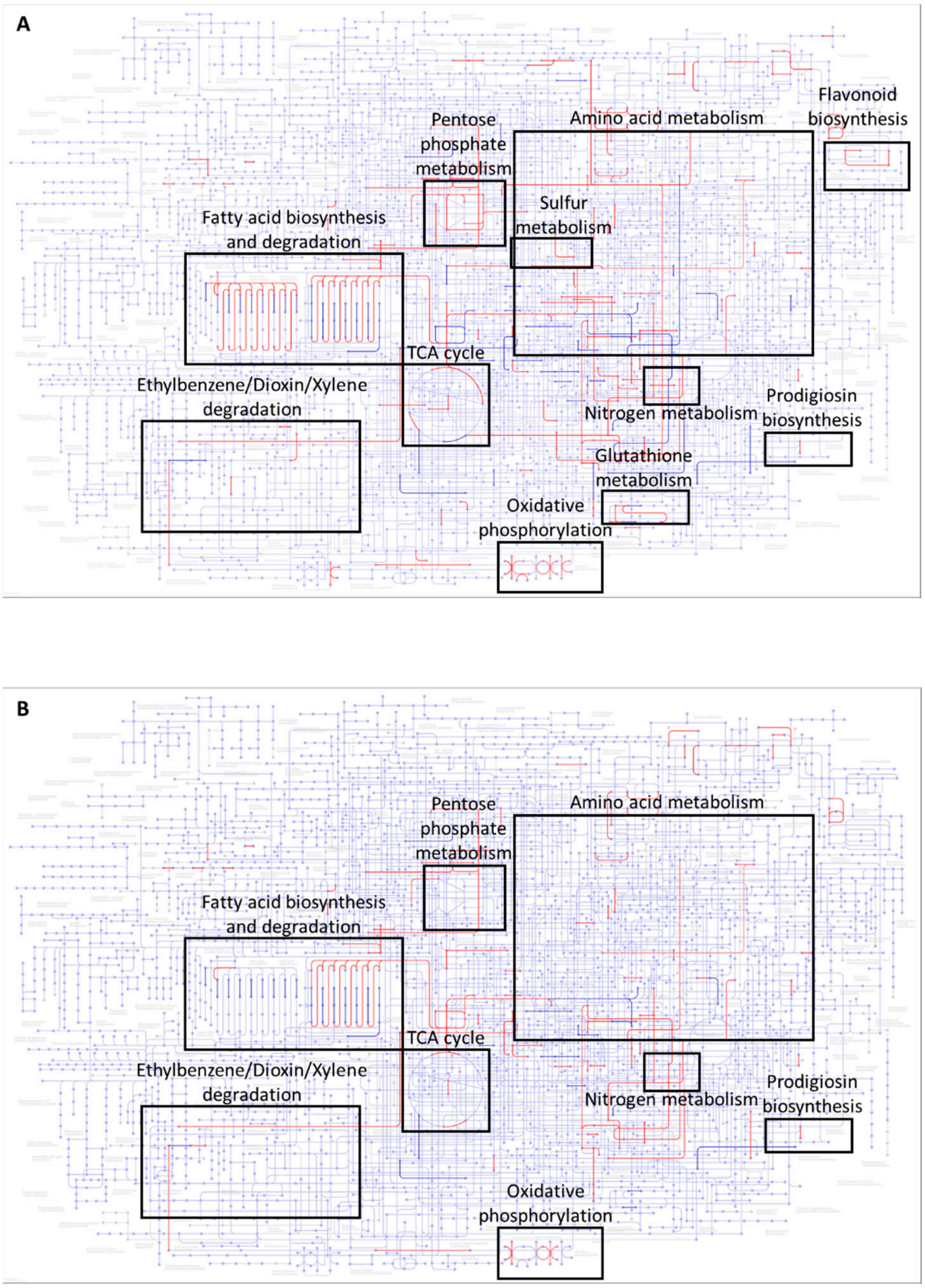

Figure S8. Prediction of metabolic pathways for (A) bacteria and (B) eukaryotes in sample NB1_S2.

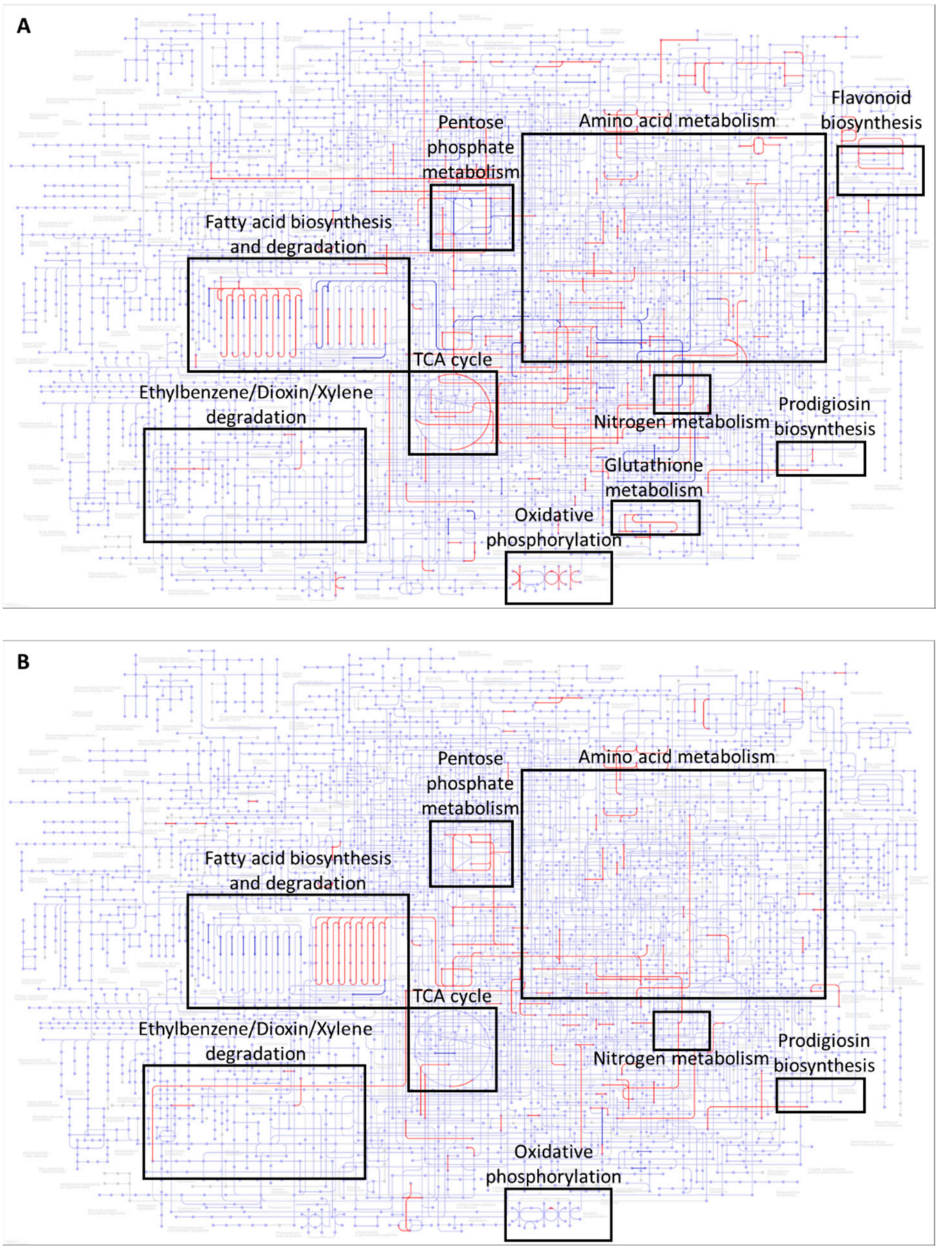

Figure S9. Prediction of metabolic pathways for (A) bacteria and (B) eukaryotes in sample DIVOL_4A .

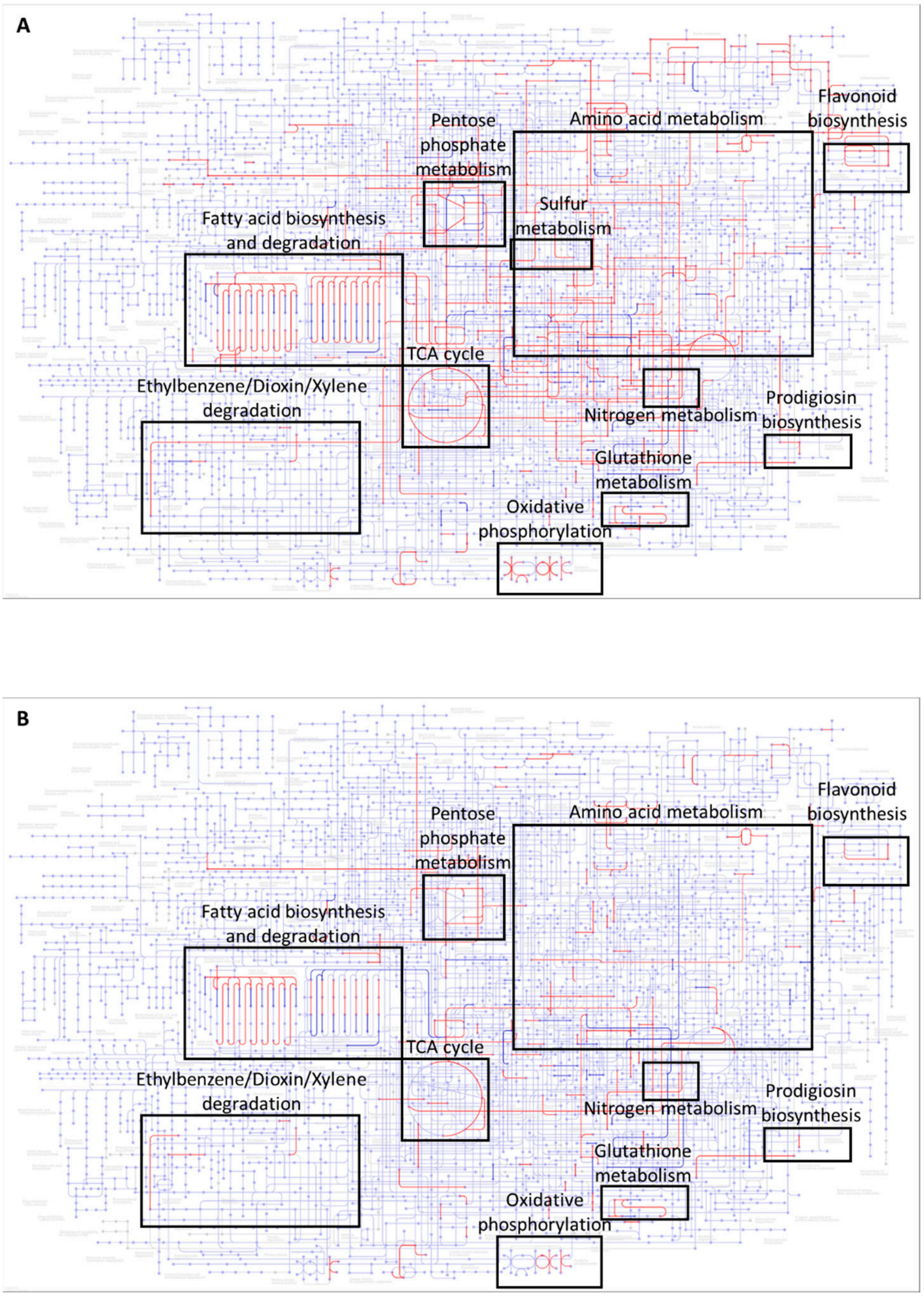

Figure S10. Prediction of metabolic pathways for (A) bacteria and (B) eukaryotes in sample DIVOL_23.
